# The diagnostic and therapeutic value of Gastrografin in small bowel obstructions

**DOI:** 10.3389/fsurg.2025.1516155

**Published:** 2025-03-12

**Authors:** Roberta Tutino, Mariachiara Cavaglià, Nicoletta Sveva Pipitone Federico, Veronica De Simone, Giacomo Deiro, Gaetano Gallo, Mauro Santarelli

**Affiliations:** ^1^ Chirurgia Generale, d’Urgenza e PS, Dipartimento di Chirurgia Generale e Specialistica, Azienda Ospedaliera Universitaria Città Della Salute e Della Scienza di Torino, Turin, Italy; ^2^ Proctology and Pelvic Floor Surgery Unit, Isola Tiberina – Gemelli Isola Hospital, Rome, Italy; ^3^ Department of Surgery, Sapienza University of Rome, Rome, Italy

**Keywords:** intestinal obstruction, small intestine, Gastrografin, diatrizoate meglumine, acute abdomen

## Abstract

**Purpose:**

Small bowel obstructions represent a major cause of hospitalization, morbidity and mortality in surgical emergency departments. The Gastrografin protocol could be an effective tool in facilitating its evaluation and treatment.

**Methods:**

A prospective observational study was conducted on patients admitted to the emergency department with a diagnosis of small bowel obstruction treated with the Gastrografin challenge to analyze risk factors related to the outcome.

**Results:**

55 patients were included. In 38 patients (69.09%) the resolution of the occlusive condition was obtained. The progression of Gastrografin in the colon at x-ray was correlated to the positive outcome (*p* = 0.001). Older (>75 years old) and frailer patients were related to protocol failure and submitted more to surgery (*p* = 0.043; *p* = 0.022). Air-fluid levels at x-ray was related to negative outcome (*P* = 0.027). Higher doses of Gastrografin (100 ml vs. 50 ml) seems unrelated to obstruction resolution. At the two-year follow-up, among the 38 patients who tested positive, 8 patients (21.05%) had further access to the emergency department due to intestinal obstruction and were re-treated conservatively.

**Conclusions:**

The standardized diagnostic-therapeutic protocol with Gastrografin is a valid tool in the non-operative management of small bowel obstructions offering a resolution of the obstructive condition in 70% of patients.

## Introduction

Small bowel obstructions (SBOs) are a significant cause of emergency hospital admission with significant morbidity and mortality rates. The classic symptomatic tetrad is represented by intermittent colicky abdominal pain, nausea associated or not with vomiting, abdominal distension and progressive closure of the bowel to feces and gas (1, 2). However, the clinical presentation may vary depending on the degree of obstruction, its location and aetiology (3–5).

The finding of fever, tachycardia, hypotension with dehydration of mucous membranes and skin may be indicative of an evolution towards sepsis (6).

The clinical management of intestinal obstruction differs from center to center and source availability.

The treatment usually involves an initial conservative therapy followed by the use of surgery in cases that do not find clinical benefit (7). Conservative management includes intravenous fluids for rehydration, nasogastric tube decompression, and medications for pain and nausea. Patients are kept fasting and clinically and laboratoristically monitored (8).

Conservative therapy is contraindicated in all cases in which there are signs of intestinal ischemia, peritonism or intestinal strangulation, persistent vomiting, the presence of an irreducible abdominal hernia or radiological signs such as free fluid in the abdomen, mesenteric edema and absence of feces in the small intestine (small bowel feces sign). In all these cases, urgent surgical treatment is indicated (9, 10). However, the conservative and surgical approach do not follow a fixed protocol in terms of both the methods and the timing and it is difficult to establish when it is necessary to change approach (11, 12).

In the absence of signs and symptoms of peritonism or ischemia, it is possible to continue with a conservative approach for 72 h but usually not more than 3–5 days (13).

Gastrografin, a hyperosmolar contrast agent, has been used to enhance the efficacy of conservative treatment, leveraging its osmotic properties (osmolarity 6 times higher than that of plasma), to draw fluid into the intestinal lumen, thereby stimulating motility and reducing edema (14–16).

Some authors have demonstrated greater effectiveness of Gastrografin compared to conventional non-operative treatment with fasting, hydration and gastric decompression (17, 18).

Moreover, the use of the Gastrografin protocol could make the evaluation of patients with intestinal obstruction more homogeneous and allow an evaluation of the effectiveness of conservative treatment vs. the decision to undertake definitive surgery (19–21).

The present study aims to evaluate the role of a standardized management protocol involving the use of Gastrografin in the treatment of patients with small bowel obstruction. In particular, we want to verify whether the adoption of this treatment protocol offers advantages in terms of resolution of the bowel occlusion, length of hospitalization, rate of surgical interventions and complications.

The primary objectives of this study were:

• To evaluate the efficacy of a Gastrografin-based conservative treatment protocol in resolving SBOs.
• To identify clinical and demographic factors that influence the success rate of this protocol, including age, comorbidities, and the presence of radiological signs indicative of more severe obstruction.

## Materials and methods

A prospective observational study was conducted at the Emergency Department, AOU Città della Salute e della Scienza, Turin, Italy. Between June 2022 and June 2023 consecutive patients presenting with a diagnosis of small bowel obstruction were included. All patients gave written informed consent.

Inclusion criteria were adult patients (aged 18 years or older) diagnosed with small bowel obstruction, based on clinical signs and symptoms (intermittent colicky abdominal pain, nausea with or without vomiting, abdominal distension, and progressive closure of stool and gas passage) and radiological findings (distension of bowel loops >3 cm in x-ray or CT scan, air-fluid levels, and absence of gas and feces in the colon) treated with the Gastrografin protocol.

Exclusion criteria were: Patients with symptoms suggestive of strangulation or ischemia, or peritonitis; abdominal or pelvic surgery within the past six weeks; patients with known abdominal or pelvic neoplasms; a history of allergy to iodinated contrast media; patients with external abdominal hernias; pregnant women.

### Gastrografin challenge

The diagnostic-therapeutic protocol with Gastrografin (Gastrografin challenge) began with hemodynamic stabilization and the placement of a nasogastric tube, maintained on gravity for 2 h. Initial abdominal x-ray was performed to confirm the diagnosis and rule out contraindications for conservative treatment. The performance of an abdominal CT scan as a second diagnostic examination was left to the discretion of each individual surgeon, should they deem it necessary. x-ray exams were performed in anteroposterior view for patients who could stand or in lateral decubitus for those who could not. The CT scan, on the other hand, was performed with intravenous iodinated contrast, except in cases of severe renal failure or allergy. Gastrografin (50–100 ml diluted in water) was administered via the nasogastric tube, which was then closed (the contrast was administered orally in patients who refused the tube). X-ray signs evaluated were air-fluid levels, intestinal loop dilatation > 3 cm, colonic dilatation. CT-scan signs were air-fluid levels, intestinal loop dilatation > 3 cm, a recognizable transition point or suspicious of adhesions as radiological report.

**Figure 1:**
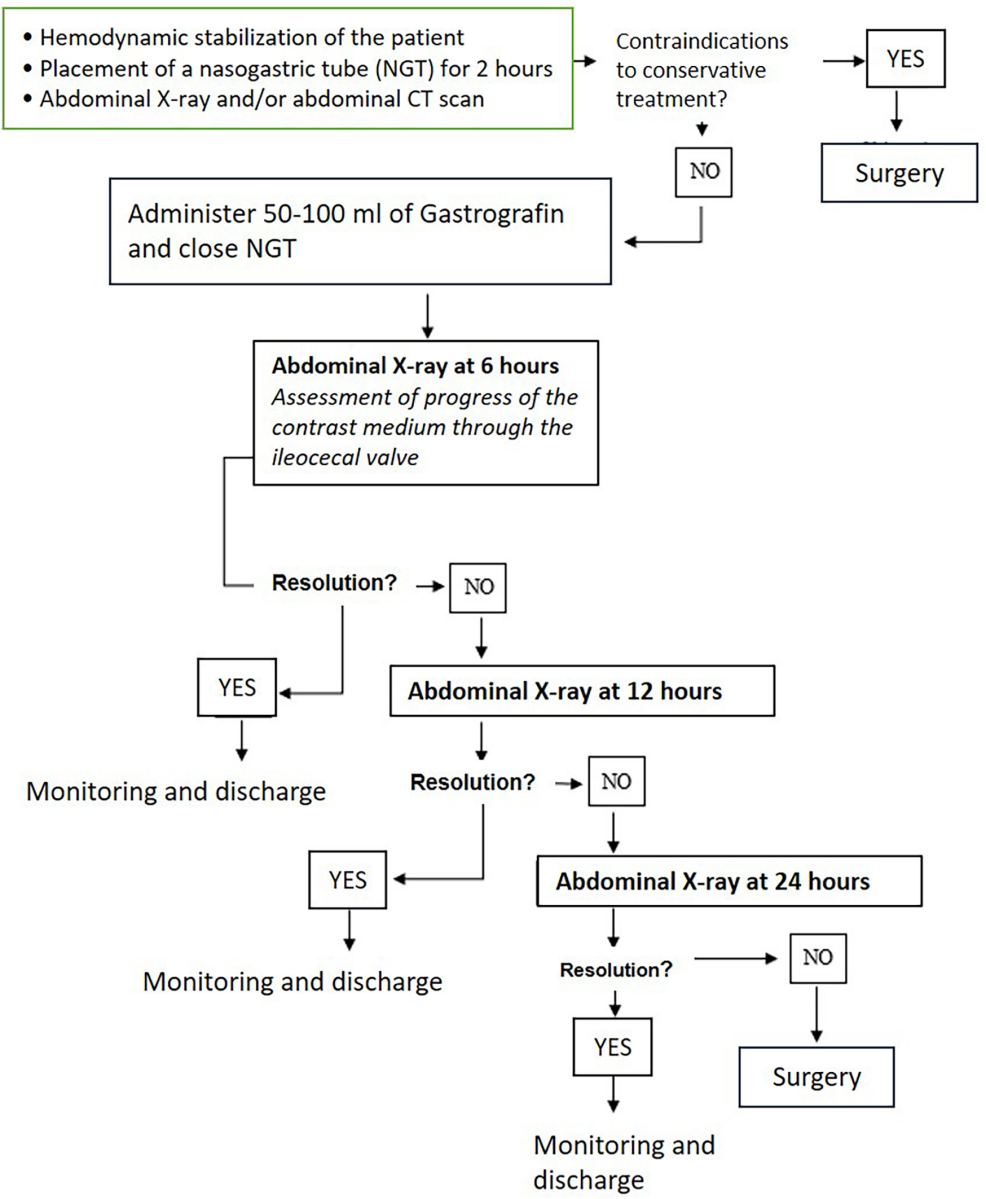
Gastrografin challenge.

Six hours post-administration, an abdominal x-ray was done to assess the contrast medium's progression. A positive outcome was defined by the contrast reaching the ileocecal valve or bowel movements resumption by stool passage. In the case of stoma patients, the presence of fecal material or contrast through the stoma was evaluated. If these conditions were not met, an x-ray follow-up was conducted at 12 and 24 h post-Gastrografin administration. Failure to progress or deterioration in clinical status indicated the need for emergency surgery Figure 1.

Demographic data, clinical history, comorbidities, laboratory results, imaging findings, and treatment outcomes were collected. The patients were divided into two groups according to the outcome of the protocol.

### Statistical analysis

Descriptive data were reported as mean ± SD, median (range) and number (percentage) of patients. The relationship between age, ASA, laboratory tests, radiological signs and protocol outcomes was analyzed using the independent-sample *t*-test or Pearson's chi-squared test. A *p*-value of <0.05 was considered to be statistically significant. Statistical analysis was conducted using GNU PSPP version 1.2.

## Results

Fifty-five patients [27 males, 28 females; mean age 68,7] diagnosed with small bowel obstruction and managed with the *Gastrografin challenge* protocol were included. Forty-six patients had a history of previous surgeries, while 9 had no surgical history.

The most common symptoms reported at presentation were abdominal pain, vomiting, and abdominal distension. Abdominal x-rays confirmed small bowel obstruction in all cases. The radiographic findings included dilated bowel loops (>3 cm) and air-fluid levels, without signs of gas or feces in the colon.

50 ml of Gastrografin was administered to 31 patients, and 100 ml to 24 patients, based on the clinical judgment, the severity of the obstruction and patient tolerance.

**Figure 2:**
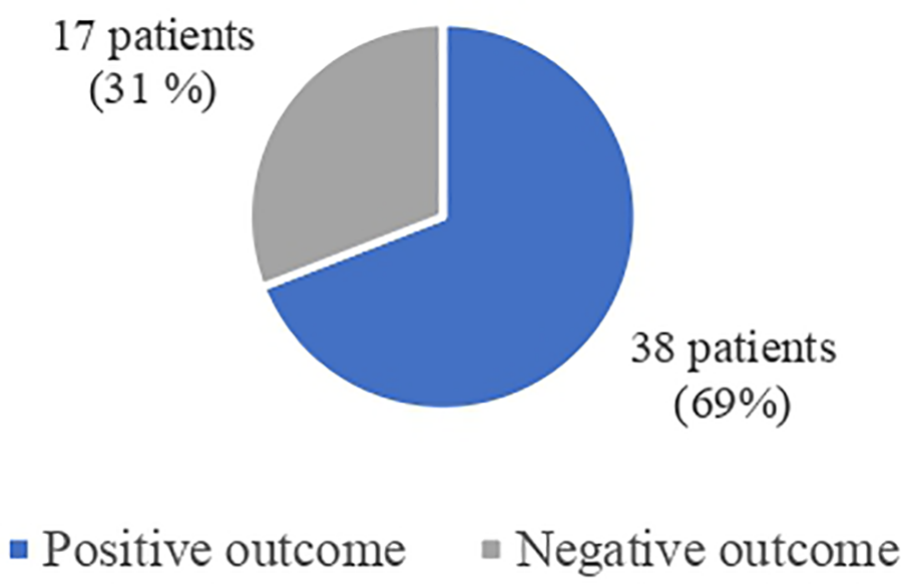
Gastrografin challenge outcomes.

In 38 patients (69.09%) the protocol had a positive outcome leading to the resolution of the bowel occlusion. Specifically, within 6 h, the contrast medium reached the colon in 29 patients (52.7%), indicating resolution of the obstruction. In 9 patients (16.3%), the contrast medium did not reach the colon at 6 h but did so by 12 or 24 h, without necessitating surgical intervention. In 17 patients the protocol had a negative outcome (30.91%), leading to surgical indication of whom 15 underwent surgery, while 2 refused further treatments (Figure 2).

Demographical data for the group with a positive and negative outcomes are presented in Table 1.

**Table 1:** Demographical and clinical data.

Demographical and clinical data	Positive outcome (38 patients)	Negative outcome (17 patients)	*P*-value
Females/Males	18/20 (32.7%/47.3%)	9/8 (53.3%/46.7%)	*p =* 0.34
Age			
– 18–39 Y.O.	5 (13.2%)	5 (13.2%)	*p* = 0.043
– 40–59 YO	4 (10.5%)	4 (10.5%)	
– 60–75 YO	19 (50%)	19 (50%)	
– 76+ YO	10 (26.3%)	10 (26.3%)	
ASA score			
– 1	9 (23.7%)	2 (13.3%)	*p* = 0.022
– 2	18 (47.4%)	3 (20%)	
– 3	9 (23.7%)	7 (46.7%)	
– 4	2 (5.3%)	3 (20%)	
Previous episodes of obstruction	16 (42.1%)	7 (33.3%)	*p* = 0.44
Previous surgeries	32 (82.8%)	14 (78.9%)	*p* = 0.73
Diagnosis of inflammatory bowel disease	2 (5.3%)	0	*p* = 0.63
History of previous neoplastic disease	19 (50%)	8 (46.7%)	*p* = 0.26
Laboratory data			
– CRP > 10.0 MG/DL	19 (50%)	9 (53.3%)	*p* = 0.98
– LEUKOCYTES > 11,000	12 (31.6%)	5 (26.7%)	*p* = 0.54
– CREATININE > 1.0 MG/DL	12 (31.6%)	11 (33.3%)	*p* = 0.86

There was no difference in terms of gender between positive and negative protocol outcomes (*p* = 0.34).

Mean age was 68,35 years old in the positive outcome and 68,34 years old in the negative outcome group. Patients older than 75-years old were respectively 52.9% and 26.3% in the negative and positive outcome groups (*p* = 0.043).

Among patients with previous surgeries (46 patients), 29 patients (63%) had a positive outcome, while 16 patients (34.8%) had a negative one (*p* = 0.74).

A previous episode of bowel obstruction was present in 14 (66.7%) of the patients that showed a positive outcome (*p* = 0.44).

There was no statistically significant difference on outcome between the population with a history of previous neoplastic disease and those without (*p* = 0.38).

There was a strong correlation between ASA scores and the need of surgery with patients with higher ASA scores more submitted to surgical intervention (*p* = 0.022) (Table 1).

Laboratory data showed no alterations in CPR, leukocytes and creatinine levels in 46.67%, 66.67% and 66.67% respectively, in the operated patients. No difference in respect to the positive outcome group were observed (*p* = 0.98; *p* = 0.54; *p* = 0.86) (Table 1).

The abdominal x-ray and CT scan signs of the two groups are showed in Table 2.

**Table 2:** Radiological findings.

Radiological findings	Positive outcome	Negative outcome	*P* value
x-ray: Air-fluid levels	27 (71.8%)	17 (100%)	*p* = 0.027
x-ray: Intestinal loop dilatation >3 cm in x-ray or CT scan	31 (81.2%)	17 (100%)	*p* = 0.08
x-ray: Colonic dilatation	3 (6.3%)	1 (7.1%)	*p* = 0.91
CT-scan evaluation	22 (57.9%)	17 (100%)	*p =* 0.01
CT scan: Air-fluid levels	28 (72.7%)	0	*p* = 0.02
CT scan: Intestinal loop dilatation	31 (80.9%)	0	*p* = 0.09
CT scan: Recognizable transition point	29 (77.3%)	0	*p* = 0.05
CT scan: Adhesions	10 (27.3%)	2 (13.3%)	*p* = 0.31

All the patients submitted to surgery previously underwent a CT-scan evaluation and 58% of the not operated ones, too.

For x-ray signs, there was no statistically significant difference between the population with intestinal loop dilation and those without regarding the likelihood of being operated on (*p* = 0.08); however, the presence of air-fluid levels on abdominal x-ray (*p* = 0.027) and colonic dilation (*p* = 0.01) correlated with the risk of surgical intervention.

For CT-scan signs, there was no statistically significant difference between the population with intestinal loop dilation and those without (*p* = 0.77), with or without a recognizable transition point (*p* = 0.91), or with or without adhesions (*p* = 0.89). However, the presence of air-fluid levels correlated with the likelihood of being operated on (*p* = 0.021).

Patients who successfully responded to the Gastrografin protocol had a significantly shorter hospital stay (median of 2 days) compared to those who required surgery (median of 24,5 days).

Among the 15 patients who required surgery, the cause of obstruction was adhesions in 13 cases and an internal abdominal hernia in 2 cases. No cases of bowel ischemia were reported in the surgical group, suggesting timely intervention.

At 2 years follow-up, among the 38 patients who had a positive result, 5 died for other than occlusive causes and 8 patients (21.05%) had a further access to the Emergency Department for episodes of occlusion of the small intestine. 7 were retreated conservatively while 1 required the placement of an endoscopic stent.

## Discussion

In this study, the Gastrografin protocol resulted in positive outcomes, resolution of the obstructive condition, in 38 out of 55 (69.09%) of the included patients.

Conservative treatment has a higher success rate in younger patients with less significant comorbidities.

Unfortunately, need for surgery increased progressively with the patient's age and ASA score, those patients who probably will benefit more from the non-operative management.

Previous surgeries and past neoplastic diseases do not correlate to the outcome of the protocol.

Similarly, the results of blood tests (particularly CRP, leukocytes, and creatinine levels), although deviations from normal values may suggest an ongoing organ function impairment, were not predictive of the therapeutic protocol's outcome.

The use of CT scan, required by some surgeons in addition to the x-ray, did not predict the success of the protocol, although performing both tests in the preliminary phase could be advantageous from a diagnostic sensitivity standpoint.

Multiple well-recognizable air-fluid levels on x-ray and/or CT scan were associated with a higher rate of surgical interventions but at the same time they weren't related to the outcome of the non-operative treatment. Surely, their presence may indicate a more severe obstructive pathology, for which conservative treatment may not be sufficient to resolve the clinical condition.

It is known that non-operative treatment with Gastrografin leads to better outcomes in resolving the obstructive condition in partial obstructions. However, the presence of air-fluid levels alone was insufficient to definitively distinguish between a partial and a complete obstruction.

The dose of Gastrografin is a source of debate. In our study, higher dose of the drug (100 ml vs. 50 ml) didn't correlate with success rate.

The average hospitalization time in the Emergency Department was 1.5 days for both operated and non-operated patients, but those undergoing surgery, had an additional 10.17 ± 1 days of post-operative hospital stay.

The advantages of the Gastrografin protocol have also been investigated by some other colleagues (Table 3).

**Table 3:** Literature data on the Gastrografin challenge.

Author year	Tipe of study	N. of patients	Control group	Positive outcome	Gastrografin group	Positive outcome	*P* value
Haule et al. 2013	RCT	50	25	64%	25	88.5%	0.001
Rahmani et al. 2013	RCT	50	23	76%	27	90.5%	0.07
Zielinski et al. 2017	RCT	316	143	56%	173	68%	0.0001
Khorshidi et al. 2019	RCT	52	26	50%	26	80.5%	0.04
Almafreji et al. 2022	/	46	/	/	46	72.5%	

In 2013, Haule et al. performed an RCT on 50 patients showing 88.5% of positive outcome with Gastrografin use in comparison to 64% in a standard conservative treatment group (*p* = 0.001) (7).

In 2017, Zielinski et al. analyzed in a RCT 316 patients, 143 were treated according to the standardized protocol with Gastrografin and 173 received conservative treatment. The success rate was 68% in the Gastrografin-treated group and 56% in the control group (*p* = 0.001) (8). Also, Khorshidi et al. demonstrated a positive outcome in 80.5% of cases, in contrast to 50% in the control group (*p* = 0.04) (17).

Recently Almafreji et al. analyzed 46 patients, all treated according to the Gastrografin protocol, showing a resolution rate of 72.2% (23).

Conversely, Rahmani et al. in a RCT didn't show a correlation to positive outcomes (90.5% in the Gastrografin group vs. 76% in the control group) (*p* = 0.07) (22).

As in our study, Desiato et al., reported no significant differences between surgical history, clinical and laboratory parameters and conservative treatment outcome. An abdominal CT scan in addition to an abdominal x-ray during the diagnostic phase should be considered based on the patient's symptoms and medical history. CT scan has excellent sensitivity in identifying high-grade SBO and can provide supplementary information about the cause and the site (24)

However, one of the focuses of the protocol is to become an instrument to use in any hospital setting.

In a recent report from Ethiopia, it emerges that CT scan is almost not performed in low-income countries. 83% of patients in their study were surgically managed and post-operative morbidity was considerable with 11.4% of SSI, 9.4% sepsis, 6.7% pneumonia, 6% reoperation and 4.7% death. The authors suggest to implement the use of Gastrografin to potentially reduce the use of surgery in SBO patients (25).

To note that conducting an abdominal CT in addition to its unavailability in some centers or areas, could also delay management times.

Ali et al. demonstrated a significant decreased length of hospital stay as well as hospital cost in early (<24 h) administration of Gastrografin, as well as a reduction in the time to surgery if a protocol is used aiding in a rapid decision-making (26).

The healthcare costs with the application of the diagnostic-therapeutic protocol with Gastrografin can be reduced since in the case of a positive outcome, surgery is avoided, reducing risks for the patients in addition to hospital stay length. In case of a negative outcome, the delay in performing surgery would be minimal, up to a maximum of 12 h.

Some limitations should be acknowledged. The study's observational nature means it cannot establish causality, and the relatively small sample size may limit the generalizability of the findings. Additionally, the study was conducted at a single center which may introduce bias related to local clinical practices and patient demographics.

In conclusion, the diagnostic-therapeutic protocol with Gastrografin should be attempted in all patients with small bowel obstruction with no clinical signs of sepsis or bowel ischemia. Older, frailer patients seem to show poorer response to conservative management. A higher dose of gastrografin did not correlate with treatment success. The long-term benefits of this approach are confirmed by the repeatability of the treatment even in subsequent admissions.

## Data Availability

The raw data supporting the conclusions of this article will be made available by the authors, without undue reservation.

## References

[1] Ten Broek RPG Krielen P Di Saverio S Coccolini F Biffl WL Ansaloni L Bologna guidelines for diagnosis and management of adhesive small bowel obstruction (ASBO): 2017 update of the evidence-based guidelines from the world society of emergency surgery ASBO working group. World J Emerg Surg. (2018) 13:24. 10.1186/s13017-018-0185-2 29946347 PMC6006983

[2] Bower KL Lollar DI Williams SL Adkins FC Luyimbazi DT Bower CE. Small bowel obstruction. Surg Clin North Am. (2018) 98(5):945–71. 10.1016/j.suc.2018.05.007 30243455

[3] Long B Robertson J Koyfman A. Emergency medicine evaluation and management of small bowel obstruction: evidence-based recommendations. J Emerg Med. (2019) 56(2):166–76. 10.1016/j.jemermed.2018.10.024 30527563

[4] Maung AA Johnson DC Piper GL Barbosa RR Rowell SE Bokhari F Evaluation and management of small-bowel obstruction: an eastern association for the surgery of trauma practice management guideline. J Trauma Acute Care Surg. (2012) 73(5 Suppl 4):S362–9. 10.1097/TA.0b013e31827019de 23114494

[5] Miller G Boman J Shrier I Gordon PH. Readmission for small-bowel obstruction in the early postoperative period: etiology and outcome. Can J Surg. (2002) 45(4):255–8. 12174978 PMC3684676

[6] Catena F Di Saverio S Coccolini F Ansaloni L De Simone B Sartelli M Adhesive small bowel adhesions obstruction: evolutions in diagnosis, management and prevention. World J Gastrointest Surg. (2016) 8(3):222–31. 10.4240/wjgs.v8.i3.222 27022449 PMC4807323

[7] Haule C Ongom PA Kimuli T. Efficacy of Gastrografin® compared with standard conservative treatment in management of adhesive small bowel obstruction at Mulago National Referral Hospital. J Clin Trials. (2013) 3(4):1000144. 10.4172/2167-0870.1000144 24729947 PMC3982137

[8] Zielinski MD Haddad NN Cullinane DC Inaba K Yeh DD Wydo S Multi-institutional, prospective, observational study comparing the Gastrografin challenge versus standard treatment in adhesive small bowel obstruction. J Trauma Acute Care Surg. (2017) 83(1):47–54. 10.1097/TA.0000000000001499 28422909

[9] Di Saverio S Birindelli A Broek RT Davies JR Mandrioli M Sallinen V. Laparoscopic adhesiolysis: not for all patients, not for all surgeons, not in all centres. Updates Surg. (2018) 70(4):557–61. 10.1007/s13304-018-0534-4 29767333 PMC6244716

[10] Loftus T Moore F VanZant E Bala T Brakenridge S Croft C A protocol for the management of adhesive small bowel obstruction. J Trauma Acute Care Surg. (2015) 78(1):13–9; discussion 19-21. 10.1097/TA.0000000000000491 25539198 PMC5125021

[11] Santillan CS. Computed tomography of small bowel obstruction. Radiol Clin North Am. (2013) 51(1):17–27. 10.1016/j.rcl.2012.09.002 23182505

[12] Jones K Mangram AJ Lebron RA Nadalo L Dunn E. Can a computed tomography scoring system predict the need for surgery in small-bowel obstruction? Am J Surg. (2007) 194(6):780–3; discussion 783-4. 10.1016/j.amjsurg.2007.09.020 18005771

[13] Keenan JE Turley RS McCoy CC Migaly J Shapiro ML Scarborough JE. Trials of nonoperative management exceeding 3 days are associated with increased morbidity in patients undergoing surgery for uncomplicated adhesive small bowel obstruction. J Trauma Acute Care Surg. (2014) 76(6):1367–72. 10.1097/TA.0000000000000246 24854302

[14] Ceresoli M Coccolini F Catena F Montori G Di Saverio S Sartelli M Water-soluble contrast agent in adhesive small bowel obstruction: a systematic review and meta-analysis of diagnostic and therapeutic value. Am J Surg. (2016) 211(6):1114–25. 10.1016/j.amjsurg.2015.06.012 26329902

[15] D'Agostino R Ali NS Leshchinskiy S Cherukuri AR Tam JK. Small bowel obstruction and the Gastrografin challenge. Abdom Radiol (NY). (2018) 43(11):2945–54. 10.1007/s00261-018-1591-3 29632988

[16] Lawrence EM Pickhardt PJ. Evaluating suspected small bowel obstruction with the water-soluble contrast challenge. Br J Radiol. (2022) 95(1130):20210791. 10.1259/bjr.20210791 34826227 PMC8822578

[17] Khorshidi HR Majidi P Pirdehghan A. Therapeutic effect of Gastrografin and predictors of operative intervention in patients with adhesive small bowel obstruction: a randomized controlled study. Turk J Surg. (2019) 35(2):131–5. 10.5578/turkjsurg.4237 32550318 PMC6796074

[18] Almafreji I Chinaka U Hussain A Lynch M Cottrell R. Role of Gastrografin in patients with small bowel obstruction. Cureus. (2020) 12(8):e9695. 10.7759/cureus.9695 32923285 PMC7486109

[19] Esaki M Tamura Y Ichijima R Suzuki S Iwamoto M Minoda Y Efficacy and timing of gastrografin administration after ileus tube insertion in patients with adhesive small bowel obstruction. Arab J Gastroenterol. (2022) 23(1):45–51. 10.1016/j.ajg.2021.12.004 35120840

[20] Khasawneh MA Ugarte ML Srvantstian B Dozois EJ Bannon MP Zielinski MD. Role of Gastrografin challenge in early postoperative small bowel obstruction. J Gastrointest Surg. (2014) 18(2):363–8. 10.1007/s11605-013-2347-6 24165871

[21] Fevang BT Jensen D Svanes K Viste A. Early operation or conservative management of patients with small bowel obstruction? Eur J Surg. (2002) 168(8-9):475–81. 10.1080/110241502321116488 12549688

[22] Cohen RB Olafson SN Krupp J Parsikia A Kaplan MJ Moran B Timing of Gastrografin administration in the management of adhesive small bowel obstruction (ASBO): does it matter? Surgery. (2021) 170(2):596–602. 10.1016/j.surg.2021.03.008 33836900

[23] Rahmani N Mohammadpour RA Khoshnood P Ahmadi A Assadpour S. Prospective evaluation of oral Gastrografin(®) in the management of postoperative adhesive small bowel obstruction. Indian J Surg. (2013) 75(3):195–9. 10.1007/s12262-012-0479-7 24426426 PMC3689375

[24] Desiato E Lucia AMA Giudici S Ammirabile A Francone M Lanza E Prognostic value of CT findings for conservative treatment failure in adhesive small bowel obstruction. Emerg Radiol. (2025) 32(1):33–40. 10.1007/s10140-024-02276-4 39073730

[25] Starr N Tadesse M Igwebuike C Sherefa K Genetu A Aregawi Y Feasibility of Gastrografin use for adhesive small bowel obstruction in low-income countries. J Surg Res. (2024) 293:239–47. 10.1016/j.jss.2023.08.017 37802018

[26] Ali M Slack DR Edmondson E Feinn R Kurtzman SH Zhang ZJ. Small bowel follow-through: treatment for small bowel obstruction or delaying the inevitable? Cureus. (2023) 15(12):e50267. 10.7759/cureus.50267 38196418 PMC10774837

